# Synthesis of ZnO/Si Hierarchical Nanowire Arrays for Photocatalyst Application

**DOI:** 10.1186/s11671-016-1803-0

**Published:** 2017-01-05

**Authors:** Dingguo Li, Xiaolan Yan, Chunhua Lin, Shengli Huang, Z. Ryan Tian, Bing He, Qianqian Yang, Binbin Yu, Xu He, Jing Li, Jiayuan Wang, Huahan Zhan, Shuping Li, Junyong Kang

**Affiliations:** 1 Fujian Provincial Key Laboratory of Semiconductors and Applications, Collaborative Innovation Center for Optoelectronic Semiconductors and Efficient Devices, Department of Physics, Xiamen University, Xiamen, 361005 China; 2 Department of Chemistry and Biochemistry, University of Arkansas, Fayetteville, AR 72701 USA; 3 State Key Lab of Silicon Materials, Zhejiang University, Hangzhou, 310027 China; 4 Fujian Provincial Key Laboratory of Eco-Industrial Green Technology, Wuyi University, Fujian, 354300 China

**Keywords:** Photocatalytic activity, Nanowire arrays, Hierarchical architecture, Semiconductor

## Abstract

ZnO/Si nanowire arrays with hierarchical architecture were synthesized by solution method with ZnO seed layer grown by atomic layer deposition and magnetron sputtering, respectively. The photocatalytic activity of the as-grown tree-like arrays was evaluated by the degradation of methylene blue under ultraviolet light at ambient temperature. The comparison of morphology, crystal structure, optical properties, and photocatalysis efficiency of the two samples in different seeding processes was conducted. It was found that the ZnO/Si nanowire arrays presented a larger surface area with better crystalline and more uniform ZnO branches on the whole sidewall of Si backbones for the seed layer by atomic layer deposition, which gained a strong light absorption as high as 98% in the ultraviolet and visible range. The samples were proven to have a potential use in photocatalyst, but suffered from photodissolution and memory effect. The mechanism of the photocatalysis was analyzed, and the stability and recycling ability were also evaluated and enhanced.

**Electronic supplementary material:**

The online version of this article (doi:10.1186/s11671-016-1803-0) contains supplementary material, which is available to authorized users.

## Background

Along with social development, the environmental pollution is becoming more and more critical. Finding a high efficient and cost-effective catalyst is one of the best ways to remove the pollutants such as organic dyes and toxic chemicals. Semiconductor photocatalysts are the key materials to complete mineralization of a wide range of dyes and organic compounds, as they offer tunable and enhanced photoresponse in ultraviolet (UV) and visible region and efficient electron-hole separation to create active radicals for photocatalysis [1]. Among the semiconductor materials studied, hybrid nanowires with different materials have been of great interest due to the potential superior efficiency coming from the unique geometry and properties, unlimited combination, and effective integration at nanoscale to implement design and material integrity [2]. In this regard, branched tree-like nanowire arrays may be the most favorite choice because they provide omnidirectional branches for effective photon absorption, enlarge surface area to react with the pollutants, maximize junction interface area for electron-hole separation, and enhance surface curvature for a high reaction kinetics [3–5].

Semiconductor materials that are currently applied in the building blocks of functional hybrid nanowire arrays includes Si [6–8], Ge [9], III–V [10–12], IV–VI [13, 14], II–VI [15, 16] and metal oxides (ZnO [2, 17–21], TiO_2_ [22], In_2_O_3_ [23], and Fe_2_O_3_ [24]). Among them, Si is the most fundamental material in current photovoltaic market and has demonstrated broad application as solar cell, sensor, and catalyst. However, the potential corrosion and high valence band maximum energy make it unsuitable alone in these fields. Another semiconductor material, ZnO, may be a good candidate to remedy these problems for its large bandgap (3.37 eV), large exciton binding energy (60 meV), and stable physical and chemical properties. Moreover, it is easily obtained in different types of nanostructures. Previously research on the ZnO/Si nanowire arrays found a very high photovoltaic current density and efficiency [17–20]. Study of the system currently concentrates on the photovoltaics [17], dye-sensitized solar cells [25], photoelectrochemical electrodes for water splitting [18, 20], and sensors [26], but literature about photocatalytic activity is rarely reported [27]. In this research, the arrays were used as a catalyst to degrade methyl red and exhibited a good photocatalytic capability. However, the arrays were grown by both chemical etching and chemical vapor deposition. Photolithography was also applied in order to fabricate patterned Si arrays in a controlled geometry and density. All these costed much time and needed sophisticated equipments. In our experiment, except the seed layer, we used solution method to grow the hybrid nanowire arrays, owning the advantages of low synthetic temperature, simple equipment, and time saving.

In the fabrication of hybrid nanowire arrays, the seed layer is a vital factor, because it decides the position, diameter, orientation, and density of the nanowire arrays. In previous research, the seed layer of the branched ZnO nanowires was respectively deposited by magnetron sputtering (MS) [18], electrospinning [25], spin coating [25], dip coating [17], as well as atomic layer deposition (ALD) [26]. All these reports are chiefly concerning on the final function of the ZnO/Si nanowire arrays. The optimization of the seed layer has yet to be resolved.

Therefore, in this article, two different seeding methods were used to figure out how it influence the growth of branched ZnO nanowires as well as the properties and function of tree-like ZnO/Si nanowire arrays. One seeding method was ALD, while the other one was MS. The photocatalytic activity of the as-grown tree-like ZnO/Si nanowire arrays was evaluated by the degradation of a typical organic dye methylene blue (MB) under UV light irradiation. The comparison of morphology, crystal structure, optical properties, and photocatalysis efficiency of the two samples was conducted. Moreover, the mechanism of the photocatalysis was analyzed, and the stability and recycling ability were also evaluated.

## Methods

### Synthesis Process

The fabrication process of ZnO/Si nanowire arrays includes substrate cleaning, wet chemical etching for Si backbones, ZnO seed layer deposition, and ZnO branches growth. First, P-type boron doped (100) Si wafers with resistivity of 1–10 Ω cm and thickness of 450 μm were used as substrates for the synthesis of the hybrid structure. The substrates were cut in a size of 10 × 15 mm^2^ and sequentially cleaned by ultrasonication in absolute toluene, acetone, ethanol, and piranha solution (H_2_SO_4_ and H_2_O_2_ in a volume ratio of 3:1) at 80 °C for 2 h, each of which was followed by ultrasonication in de-ionized water.

Second, after the substrates were dried with N_2_ flow, they were immersed in aqueous solution of 4 M HF and 0.02 M AgNO_3_ in a Teflon vessel for a galvanic displacement reaction at 50 °C for 30 min. The post-etched substrates were transferred to the solution of HCl/HNO_3_/H_2_O in a volume ratio of 1:1:1 overnight to remove the reduced Ag nanoparticles during the chemical etching. The substrates were then thoroughly rinsed with de-ionized water and dried in air.

Third, the substrates were divided into two equal groups to grow ZnO seed layer by applying the two different methods. One group, denoted as sample ALD, was deposited using TALD-100A ALD system (Keming Co. Ltd., China), while the other one, denoted as sample MS, was grown using JC500-3/D MS system (Chaomai Co. Ltd., China). In detail, to make sample ALD, the reactive chamber of the ALD system was preheated in nitrogen atmosphere at 100 °C for 24 h, then the substrates were placed into the chamber. Afterwards, the pressure of the chamber was pumped to 0.15 torr followed by heated to 150 °C. The 167 cycles of Zn(C_2_H_5_)_2_ dose for 0.02 s, N_2_ flow for 25 s, water vapor dose for 0.015 s, and N_2_ flow for 25 s were required to grow a ZnO seed layer with 30 nm thickness. As for sample MS, the substrates were transferred into a MS chamber in argon carrier gas. The background pressure was evacuated to be 10^−4^ Pa, and the working pressure was set at 1 Pa. The ZnO target was pre-sputtered for 10 min to gain a clean surface, then the seed layer was deposited on the substrates for 3 min at room temperature with sputtering power of 80 W. The film thickness was characterized to be about 30 nm by a planar Si substrate.

Fourth, ZnO nanowires were synthesized by hydrothermal method. A 500-ml aqueous solution of 25 mM Zn(CH_3_COO)_2_.2H_2_O and 25 mM C_6_H_12_N_4_ was filled into a glass beaker, which was agitated and heated in a magnetic stirring apparatus. After the temperature kept stable at 90 °C, the seeded substrates were soaked vertically in the solution for a period of 40 min. The as-grown samples were removed from the solution and rinsed with de-ionized water several times, then they were annealed at 420 °C for 30 min in an oven to obtain ZnO nanowires with better crystallinity.

### Characterization

Morphology of the as-prepared samples was characterized by a ZEISS Sigma scanning electron microscopy (SEM) with an accelerating voltage of 15.0 kV. Chemical composition of the seeded substrates was analyzed using an energy dispersive X-ray (EDX) spectroscopy as attached on the SEM. Structural quality of the nanowire arrays was evaluated by an X’Pert PRO X-ray diffraction (XRD) with Cu Kα radiation (λ = 1.54056 Å). Photoluminescence (PL) spectra were collected on a Hitachi F-7000 fluorescence spectrophotometer with an excitation wavelength of 325 nm. The parameters of PMT voltage, scan speed, and slit width were set at 700 V, 240 nm/min, and 5.0 nm for all the samples. Diffuse reflection spectra and absorption spectra were taken on an Agilent Carry-5000 UV-Vis-NIR spectrophotometer. X-ray photoelectron spectroscopy (XPS) was recorded in a PHI Quantum 2000 with Al K_α_ X-ray excitation source (*hv* = 1486.6 eV). All the measurements were carried out at room temperature in normal conditions.

### Photocatalytic Degradation

The photocatalytic activity of the nanowire arrays was evaluated by the degradation of MB dye under UV light irradiation at ambient temperature. Aqueous suspension of MB (1 × 10^−5^ M) in a volume of 20 ml was poured into a glass petri dish with a diameter of 85 mm, then a piece of sample was immersed in the solution with the nanowires facing upwards. They were subjected to UV light in an intensity of ~4.5 mW/cm^2^. The photodegradation was performed 1 h per time, and three successive reaction periods on the MB solution with identical concentration were conducted for each sample. The photocatalytic efficiency was analyzed by measuring absorption spectra of the degraded MB solution.

For the enhancement of photocatalytic activity and recycling ability, the surface of sample ALD was coated by a layer of Ag particles and TiO_2_ film. The Ag particles were reduced by immersing the sample in a suspension of Na_3_C_6_H_5_O_7_.2H_2_O and AgNO_3_ in a mole ratio of 20/2 mM in 100 mL de-ionized water, then irradiated by sun light in an intensity of ~23.5 mW/cm^2^ for an hour [28]. The sample was rinsed several times in de-ionized water and dried in air. Afterwards, a layer of TiO_2_ film with 10 nm thickness was deposited on it by ALD. The sample was finally annealed at 400 °C in nitrogen atmosphere for 3 min in an oven.

## Results and Discussion

### Morphology and Crystal Structure

**Fig. 1:**
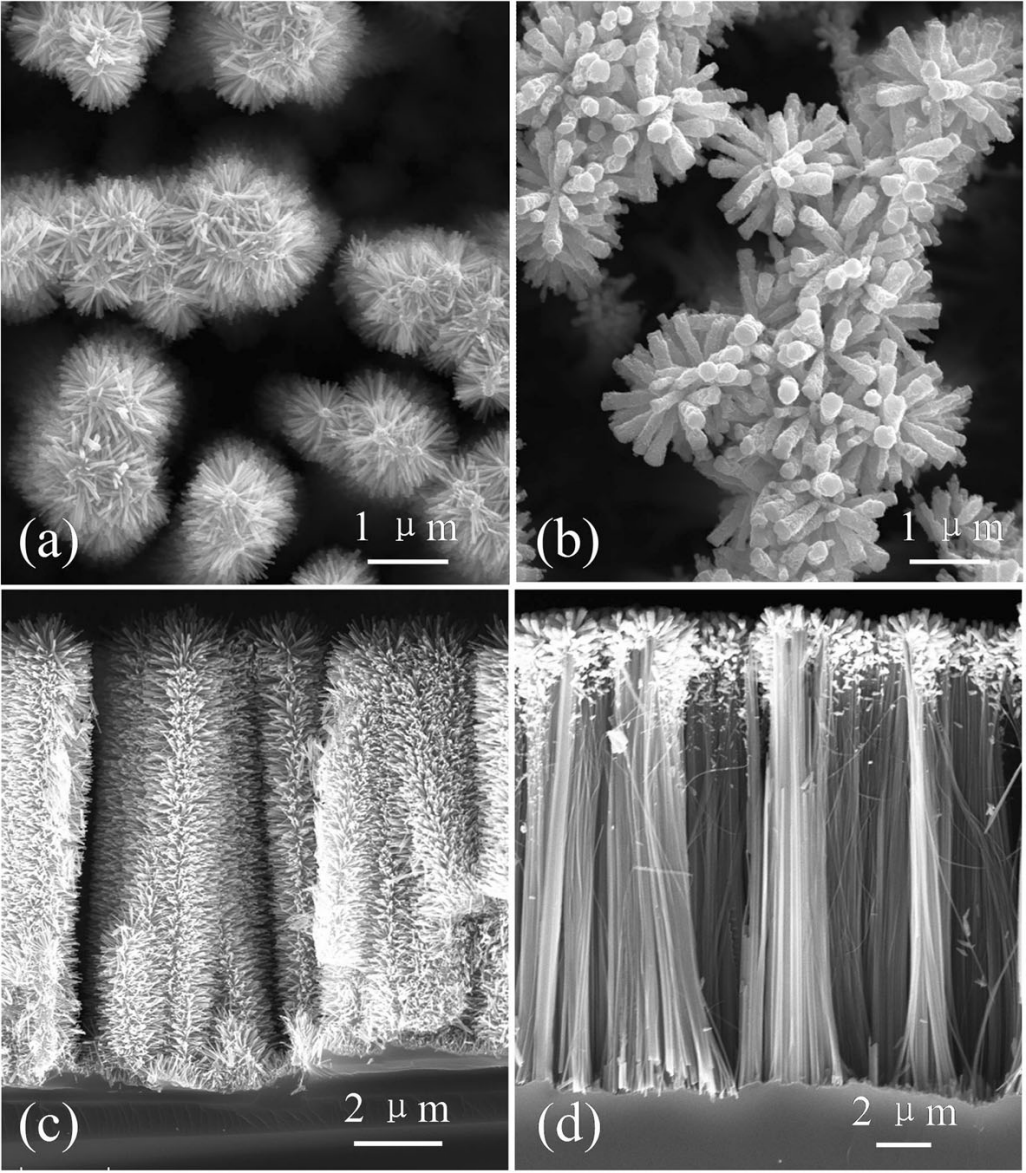
Top view (*top row*) and cross-sectional view (*bottom row*) SEM images of ZnO/Si nanowire arrays of sample ALD (**a**, **c**) and sample MS (**b**, **d**)

Figure 1 shows the SEM images of sample ALD and sample MS after hydrothermal growth. The top view and cross-sectional view of sample ALD (Fig. 1a, c) reveal that ZnO nanowires uniformly grow along the normal direction on the surface of Si nanowires and even in the bottom between Si nanowires. The length of the ZnO nanowires is 500 nm approximately, while their diameter is in a limited range of 30–50 nm. They own a smooth surface, which suggests good crystallinity and uniform crystal orientation, and is beneficial to the conduction of the injected electrons and may enhance the photocatalytic performances. However, for sample MS (Fig. 1b, d), the ZnO nanowires only stand on the top part of Si nanowires. There is a clear variation in diameter and length of ZnO nanowires in different positions. For the tip area of Si nanowires, the ZnO nanowires are about 1 μm in length and 100–200 nm in diameter, exhibiting a greater magnitude as compared with sample ALD. For the area below the tip end, lots of ZnO nanowires are also observed with smaller size. All these cause a non-uniformity of distribution, length, and diameter of ZnO branches on the Si backbones. Moreover, the ZnO nanowires are full of potholes that result in a rough surface. It is worthwhile to point out that the Si nanowire backbones have the similar structure and dimension for the two kinds of specimens. They are about 16 μm in length and random in diameter due to the nature of metal-assisted electroless etching method [29]. The etched nanowires possess a quite clean and smooth surface without any holes or reduced Ag particles, as observed in Fig. 1d.

**Fig. 2:**
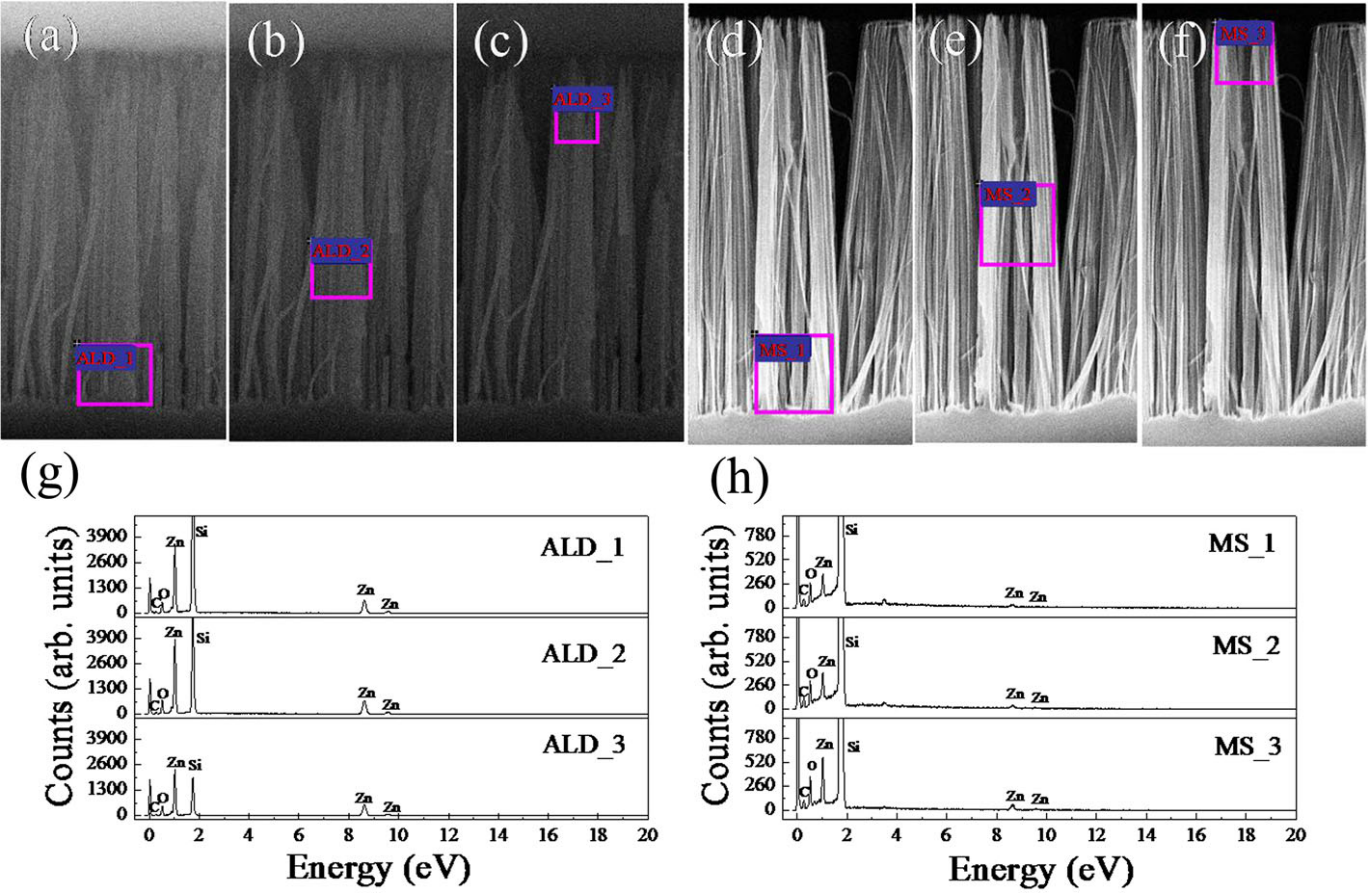
EDX spectra of Si nanowire arrays with seed layer at different positions of sample ALD ( **a**–**c**, **g**) and sample MS (**d**–**f**, **h**)

**Fig. 3:**
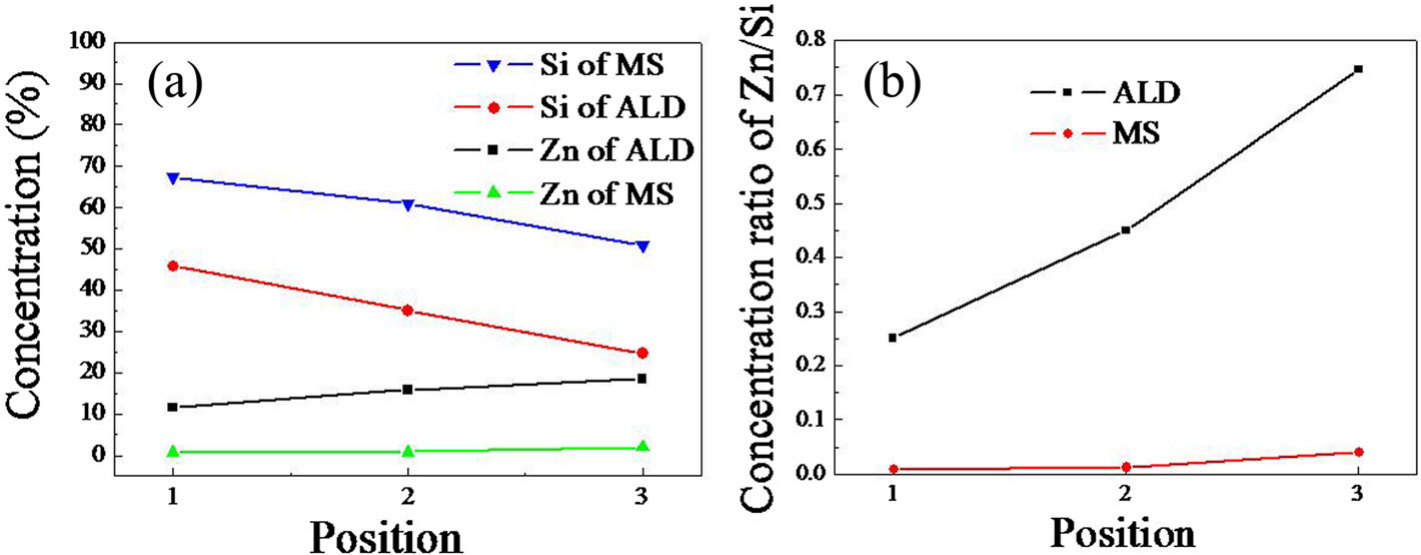
Concentration variation of elements of Si and Zn (**a**) and Zn/Si ratio (**b**) of seeded sample ALD and sample MS at different positions in Fig. 2

It has been conjectured that the non-uniform distribution of ZnO nanowires on Si backbones is due to the non-uniformity of Si nanowire diameters, large surface curvature of the Si nanowires, and uneven coating of the ZnO seeding layer as well as the damage during the sample preparation [18, 20]. In order to verify this, EDS spectra of sample ALD and sample MS after seeding process were performed at different positions, as shown in Fig. 2. Except the element C that was reduced from the reagents in the hydrothermal growth, only the signals of O, Zn, and Si elements are detected without any reduced Ag or other impurities. This is further corroborated by the following XRD and XPS analysis wherein no Ag peak is observed in the specimens. The variation of elements Si and Zn and Zn/Si ratio of the two samples as calculated from Fig. 2 are depicted in Fig. 3. In the detected region, the Zn atoms of seeded sample ALD give a concentration increasing from the bottom 11.51% through the middle 15.75% to the top 18.41%, while those of seeded sample MS chiefly centralize on the top of Si nanowires and barely come to the bottom. Their concentration is only 0.65% in the bottom and gets 2.08% on the top. The detected concentration of Si atoms is much larger than that of Zn atoms, especially for sample MS. Therefore, the Zn/Si ratio in the identical positions is obviously different for the two specimens. It increases from the bottom 0.251 to the top 0.747 for sample ALD, while it rises from 0.00966 to 0.0409 for sample MS, as shown in Fig. 3b. These distribution differences can be well attributed to the film deposition methods. The ALD apparatus deposit films layer by layer with high uniformity and conformality, even on high aspect ratio substrates. Though MS apparatus can deposit films rapidly, it is not good at film growth on substrates with high curvature effect. The distinct morphology difference of the two kinds of samples may induce unlike properties and performances.

**Fig. 4:**
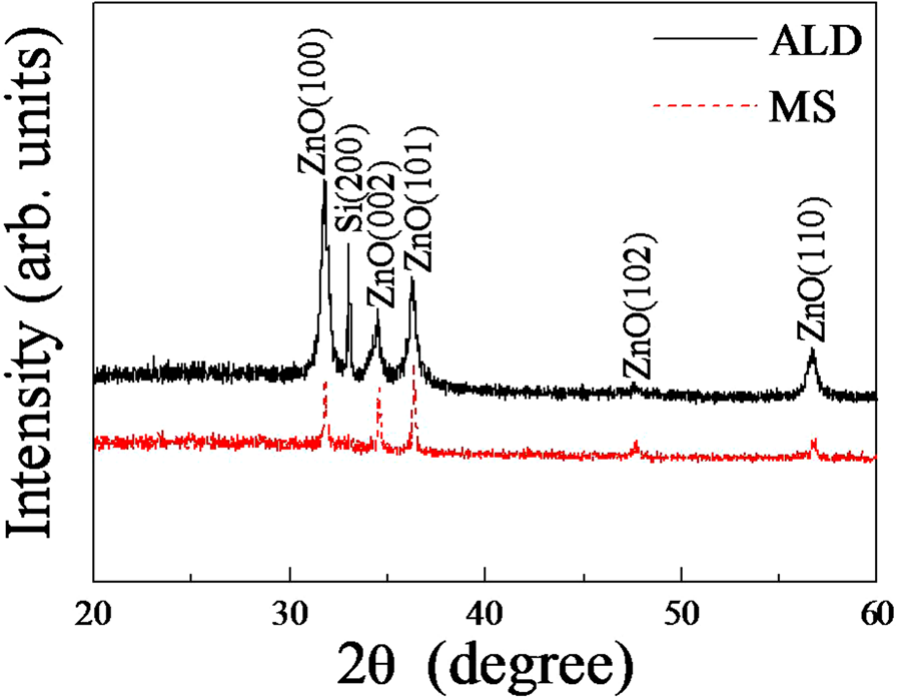
XRD patterns of sample ALD and sample MS. The ALD curve was shifted upward in contrast to MS curve for comparison

Figure 4 shows the XRD patterns of sample ALD and sample MS. The observed peaks are well indexed to ZnO crystallographic phase with hexagonal wurtzite crystal structure (JCPDS no. 36-1451). There is an additional diffraction peak corresponding to Si(200) in the pattern of sample ALD, which may result from the exposure of Si nanowire backbones or thin thickness of ZnO film. No extra peaks relating to any impurity or Ag particles are observed, which confirms that the as-grown products are pure ZnO/Si hybrid nanostructures. The diffraction peaks of sample MS are sharper than those of sample ALD, revealing bigger mean size of sample MS crystalline grains on the basis of Debye–Scherrer formula [30]. However, as the peaks intensity of sample MS is weaker than or equal to that of sample ALD, it cannot deduce that sample MS has a better crystallinity than sample ALD from the XRD patterns. In fact, the crystallinity of sample ALD is better than that of sample MS, as obviously seen in SEM images in Fig. 1 and the following PL spectra.

### Photoluminescence and Optical Reflection

**Fig. 5:**
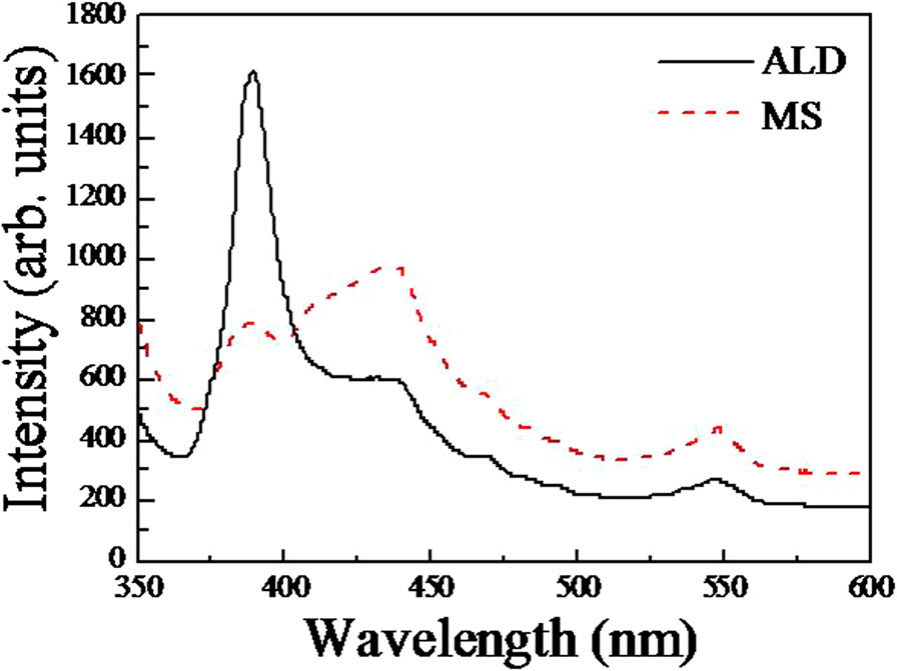
PL spectra of sample ALD and sample MS. The curves were both median smoothed (7 points) and Savitsky–Golay smoothed (3rd order, 7 points) automatically in the same measuring conditions

PL spectra of the two samples are displayed in Fig. 5. Three peaks are obviously observed in the spectrum with wavelength positions of 388, 435, and 548 nm. The first peak at 388 nm corresponds to the band edge luminescence of ZnO crystal [31], which is red-shifted from the bandgap of bulk ZnO (368 nm, 3.37 eV). The reason may be multifold, including the Si component in the hybrid nanostructure, the growing method with especial reagents, and the existence of oxygen vacancy in ZnO nanowires [32] and carbon species as established in EDS spectra. The bandgap reduction suggests that the arrays can absorb much light in a far wider wavelength, which is favorable for photocatalytic and photoelectric performances. The second and third peaks are ascribed to Zn interstitial atoms [33] and O vacancies [34]. This is an another evidence of better crystallinity of sample ALD compared with sample MS, as the characteristic peak originating from band edge emission is much stronger, and the extra peaks corresponding to the defects are weaker for the former than those for the latter.

**Fig. 6:**
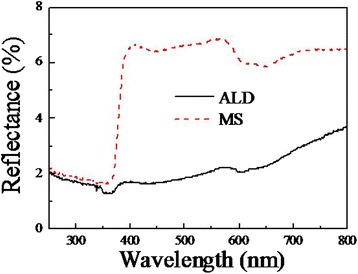
Diffuse reflection spectra of sample ALD and sample MS

The reflection spectra of the specimens are shown in Fig. 6. They own a low reflectance across the entire UV and visible range. For sample ALD, the reflectance is lower than 3.7% in the measured wavelengths between 250 and 800 nm, Most of it is stable at 2%. As the substrate is opaque, this means that all the other light is absorbed by the specimen. Therefore, the light absorption of sample ALD is as high as 98%, which may result from synergistic band structure of the heterogeneous materials, the light trapping effect for the hierarchical architecture, and the smooth transition of refractive index from air (*n* = 1) through ZnO branches (*n* = 2.0) to Si backbones (*n* = 3.5). There is no significant difference between the two curves in UV region. However, for sample MS, the reflectance gives a sharp jump at the wavelengths between 360 and 400 nm and is 2 to 3 times larger than that of sample ALD in visible region. The optical bandgap estimated from the jump with Tauc model [32] is about 388 nm, which agrees well with the result of PL spectra in Fig. 5, as both are due to the electron transition from the valence band to the conduction band of ZnO. The stronger absorption of sample ALD than that of sample MS in the visible region may be assigned to larger total surface area and better morphology of ZnO nanowires that uniformly grow on the sidewall of Si nanowires, as obviously seen in Fig. 1.

### Photocatalytic Performances

**Fig. 7:**
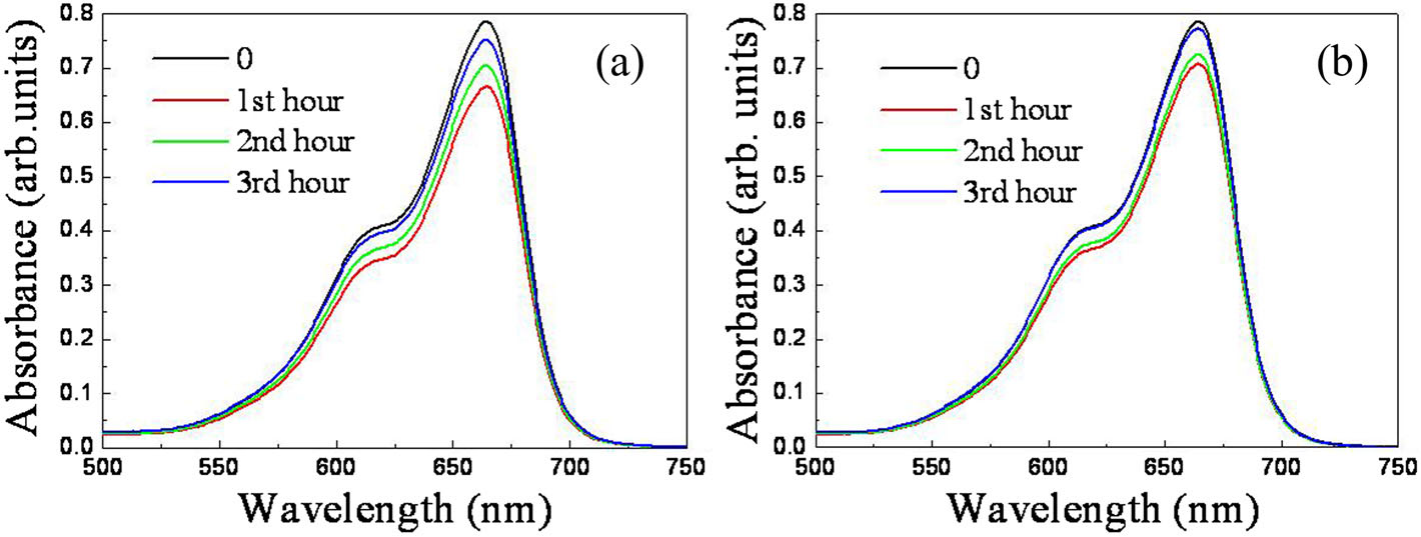
Absorption spectra of degraded MB aqueous solutions as catalyzed by sample ALD (**a**) and sample MS (**b**) under irradiation of UV light, in which 0, 1st, 2nd, and 3rd hours represent spectrum of MB solution before photodegradation, after photodegradation in the 1st, 2nd, and 3rd hours, respectively

To measure the photocatalytic activity of the two samples, time-dependent photodegradation of MB solution was conducted. When there was no ZnO/Si nanowire arrays in the solution, the MB dye was stable and no obvious degradation was observed under irradiation of the UV light, suggesting that the photoinduced self-sensitized photodegradation of MB was negligible. Figure 7 shows the absorption spectra of MB aqueous solutions as catalyzed by sample ALD and sample MS. The peak intensity of the post-degradation MB solution is lower than that of original solution, suggesting photocatalytic activity of the two specimens. However, the photocatalytic efficiency is decreased with an extended period. The relationship between the photocatalytic efficiency and the time period is calculated. For the MB solution, the absorption intensity at the peak position of ~665 nm can be characterized,

1 $$ T=76179.87C+0.01155, $$

where *T* is the absorbance and *C* is the concentration. The efficiency of each degradation is obtained by the following formula,

2 $$ E=\left(1-C/{C}_0\right)\times 100\%, $$

where *E* is the photocatalytic efficiency, *C* is the concentration of post-degradation MB solution, and C_0_ is the concentration of an original MB solution. The calculated efficiencies are presented in Table 1. In the first catalysis cycle, sample ALD demonstrates an efficiency of 15.26%. The efficiency of the following cycles decreases quickly. The photocatalytic efficiency of sample MS presents the same tendency. However, its efficiency is always lower than that of sample ALD. As the photocatalytic performance of nanomaterials is significantly influenced by different parameters, including bandgap, morphology, surface area, particle size, crystallinity, and surface defect, in contrast to the structural and optical properties of the two samples, sample ALD owns a larger surface area and surface curvature effect, better crystallinity, and higher absorption, which leads to more active sites, easier mass transportation, faster rate of reactions, and improved dye mineralization efficiency.

**Table 1:** Photodegradation efficiency of MB solution as catalyzed by sample ALD, sample MS and Si nanowire arrays in Figs. 7, 8, and 13

Time	Sample ALD	Sample MS	Si nanowires	Sample ALD in coating layer
1st hour	15.26%	9.93%	12.33%	11.52%
2nd hour	10.36%	7.75%	11.15%	11.47%
3rd hour	4.34%	1.54%	7.22%	10.96%

**Fig. 8:**
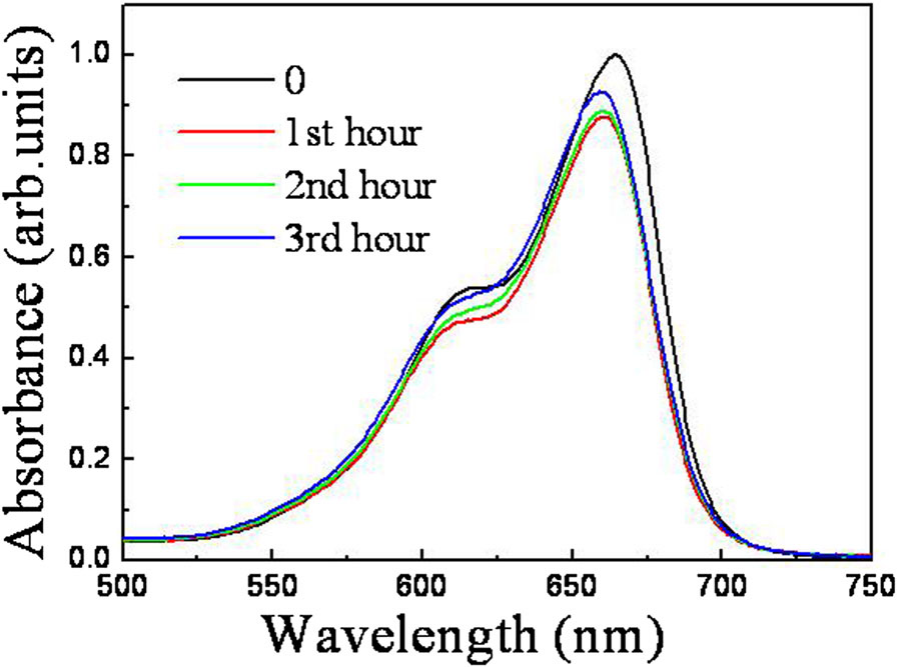
Absorption spectra of degraded MB aqueous solutions as catalyzed by silicon nanowire arrays under irradiation of UV light, in which 0, 1st, 2nd, and 3rd hours represent spectrum of MB solution before photodegradation, after photodegradation in the 1st, 2nd, and 3rd hours, respectively

For comparison, the photodegradation of MB dye in Si nanowire arrays was also carried out. Figure 8 presents the absorption spectra of MB aqueous solutions as catalyzed by Si nanowire arrays under UV light. Besides a blue shift of the absorption peak, the spectral intensity of the MB solution is also reduced after photodegradation in the catalyst, indicating the photocatalytic ability of the specimen. The photocatalytic efficiency is also calculated and shown in Table 1. The efficiency of the specimen decreases in the same way as those hybrid nanowire arrays, but the decreasing rate is slower than the others. It reduces only ~5% in the 3 h, while the others reduce nearly twice as much. Moreover, the efficiency of the Si nanowire arrays in the first hour is in between those of sample ALD and sample MS. The efficiency difference may be primarily ascribed to the structural characteristic of each sample. For sample MS, the branched ZnO nanowires mainly grow on the top of Si backbones. The long branches (1 μm) cross together, which blocks the transportation of MB dye from the top to the bottom, and only supplies the active sites on the top. However, for sample ALD, the branched ZnO nanowires conformally grow on the whole sidewall of the Si nanowire. The length of the branches is only 500 nm, which leaves enough gap for the dye transportation. Therefore, in addition to the p–n junction that promotes the separation of photoinduced charge carriers and lowers electron-hole recombination, sample ALD provides abundant surface states for the photodegradation, resulting in the highest efficiency.

### Photocatalytic Mechanisms

**Fig. 9:**
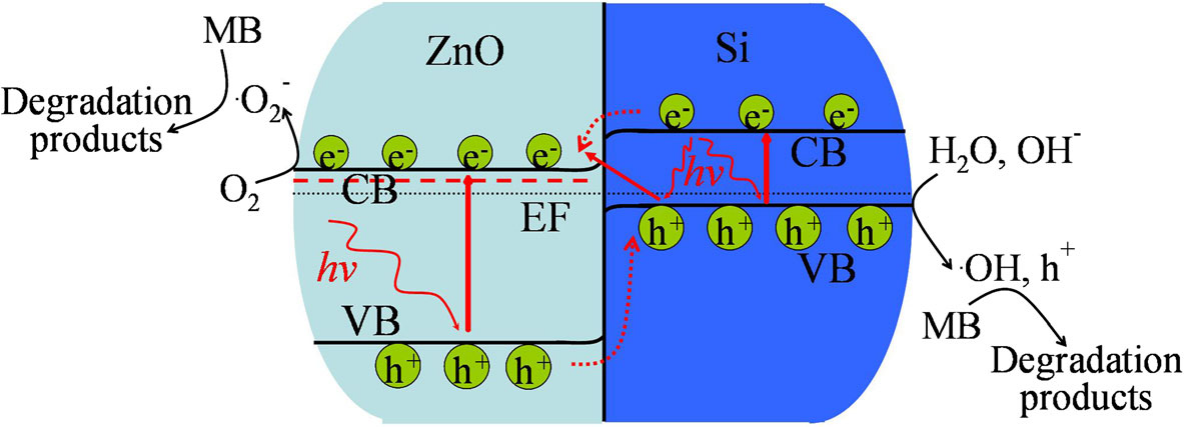
Schematic energy band diagram and proposed photocatalytic mechanisms of the ZnO/Si nanowire arrays under UV light irradiation

It has been confirmed that the photodegradation of organic dye pollutants in the semiconductor catalysts is due to the presence of active species, including hydroxyl radical ⋅ *OH*, photogenerated hole *h* ^+^, and superoxide anion radical $\cdot {O}_2^{-}$ [35]. According to the obtained results, the mechanisms underlying the photocatalytic activity of tree-like ZnO/Si nanowire arrays towards the degradation of MB dye may be described as follows and be schematically illustrated in Fig. 9. The UV light induces electron-hole pair separation in the valence bands of ZnO and Si, and excites the electrons to their conduction bands. As the conduction and valence band edges of ZnO branches straddle those of p-type Si backbones [36], and the conduction band of ZnO is lower than that of Si, the free electrons in the conduction band of Si prefer to flow to the conduction band of ZnO. Moreover, some part of electrons may also directly jump from the valence band of Si to the conduction band of ZnO in the interface of the heterojunction. The photogenerated electrons in the conduction bands of ZnO are captured by oxygen molecules that adsorbed on the ZnO surface to generate $\cdot {O}_2^{-}$ species, while the holes in the valence bands of Si react with water molecules or hydroxide anion in the solution to produce ⋅ *OH* and *h* ^+^ species, both of which cause the decomposition of organic dye molecules. The oxygen vacancies and carbon species in the nanotrees may also enhance the photocatalytic performance as they can serve as electron traps for the fast capture of photoexcited electrons, which forbids the recombination of photogenerated electrons and holes. It’s conjectured that the $\cdot {O}_2^{-}$ species in the system may play a dominant role in the degradation of MB dye, as the nanowires provide direct path for the electron transportation and enough surface area in the tree branches to contact with the dye molecules. This can be detected by adding scavengers in the solution and will be studied in our further experiment.

### Photochemical Stability

**Fig. 10:**
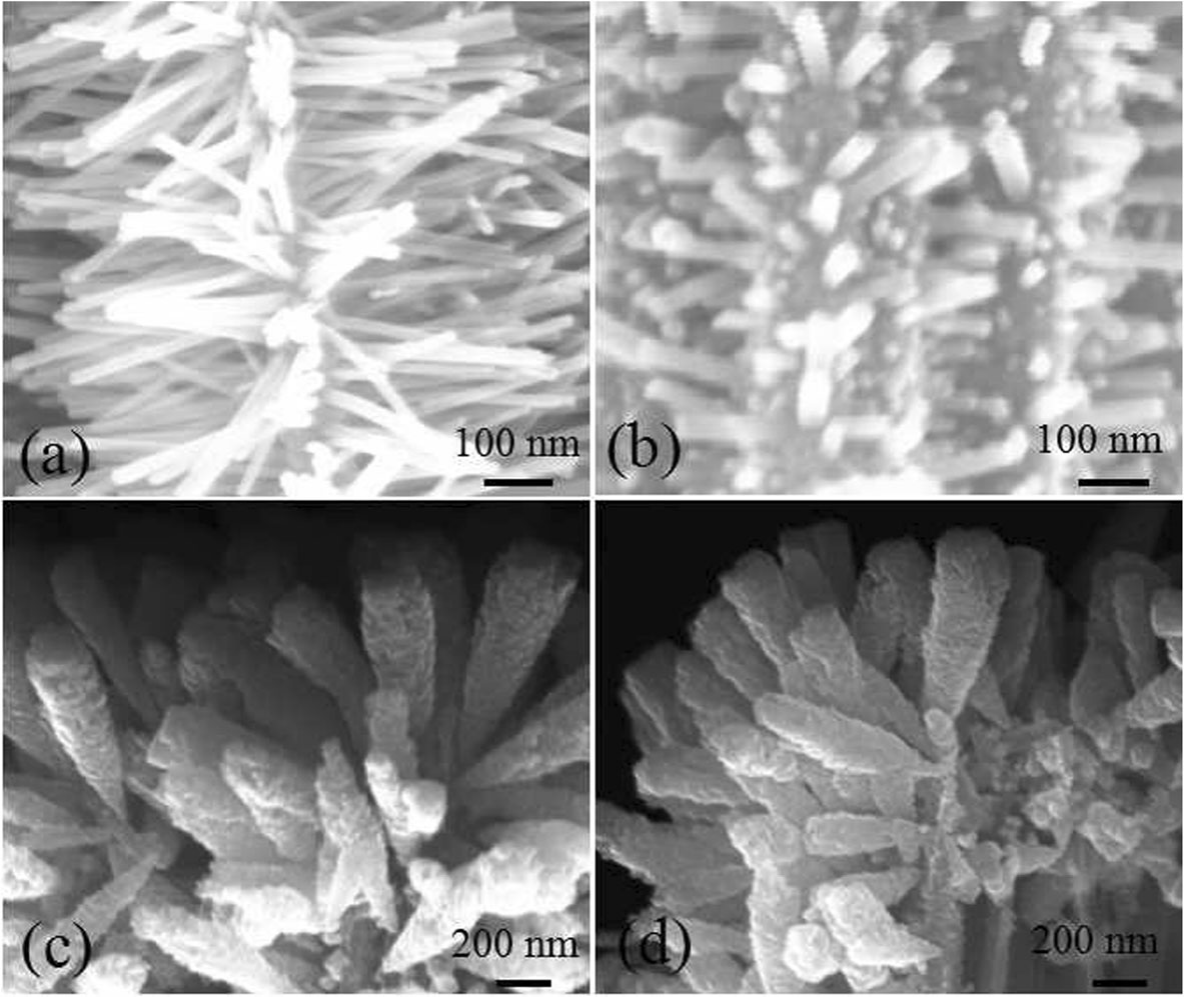
Cross-sectional view SEM images of sample ALD (**a**, **b**) and sample MS (**c**, **d**) before and after photocatalytic performance

As we can see in Fig. 7a, b, the photocatalytic efficiency of both the samples decreases with increasing irradiation period. There may be two reasons for the phenomena. One is the dissolution of ZnO/Si nanowires in the process of photocatalysis under UV light, the other is the inactive surface as the illuminated area becomes less negatively charged during the photocatalytic process when the positive holes are drawn to illuminated area by the space charge layer [37]. The function for the latter case can be recovered as a memory effect after a dark night interval. Figure 10 shows the cross-sectional SEM images of sample ALD and sample MS before and after photocatalysis. For sample ALD in Fig. 10a, b, the branched ZnO nanowires become sparse, short, and massive after photocatalysis. But for sample MS in Fig. 10c, d, the change is not so obvious. The ZnO branches seem to keep stable after the photocatalysis. Therefore, the dissolution of ZnO branches may play a critical role in the declining catalytic effect of sample ALD, whereas the inactive surface for the charge loss is a vital factor affecting the catalytic effect of sample MS. This difference is also corroborated by XPS spectra (see Additional file 1). The intensity under the deconvoluted peaks related to the Zn bond of sample ALD is reduced after the photocatalytic performance, while that of sample MS is enhanced in the process. As the intensity responds to the amount of the binding bond, the decreasing intensity indicates the loss of ZnO in sample ALD, whereas the enhancing intensity demonstrates the rise or stable state of ZnO in sample MS.

**Fig. 11:**
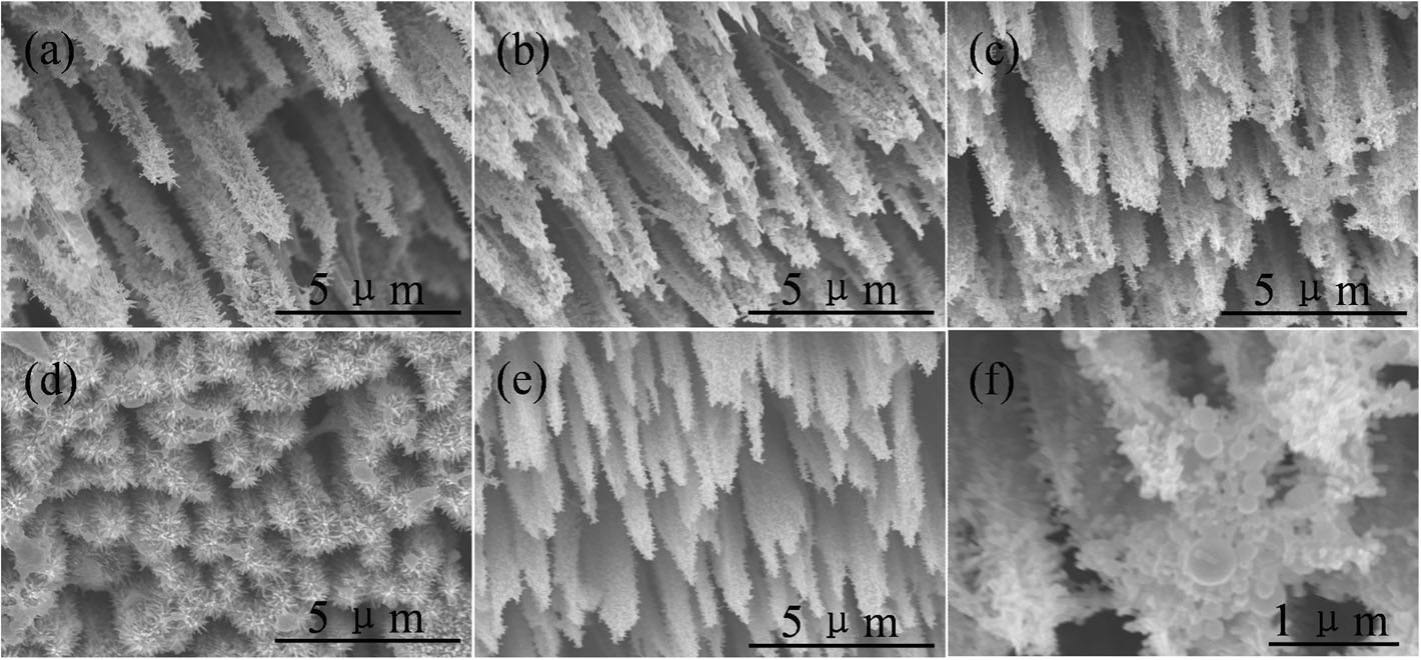
SEM images of sample ALD with and without the coating layer. **a** Pristine trees. **b** Trees coated by TiO_2_. **c** Trees coated by Ag. **d** Trees coated by Ag/TiO_2_. **e** Trees coated by TiO_2_/Ag. **f** Amplified image of trees coated by Ag

**Fig. 12:**
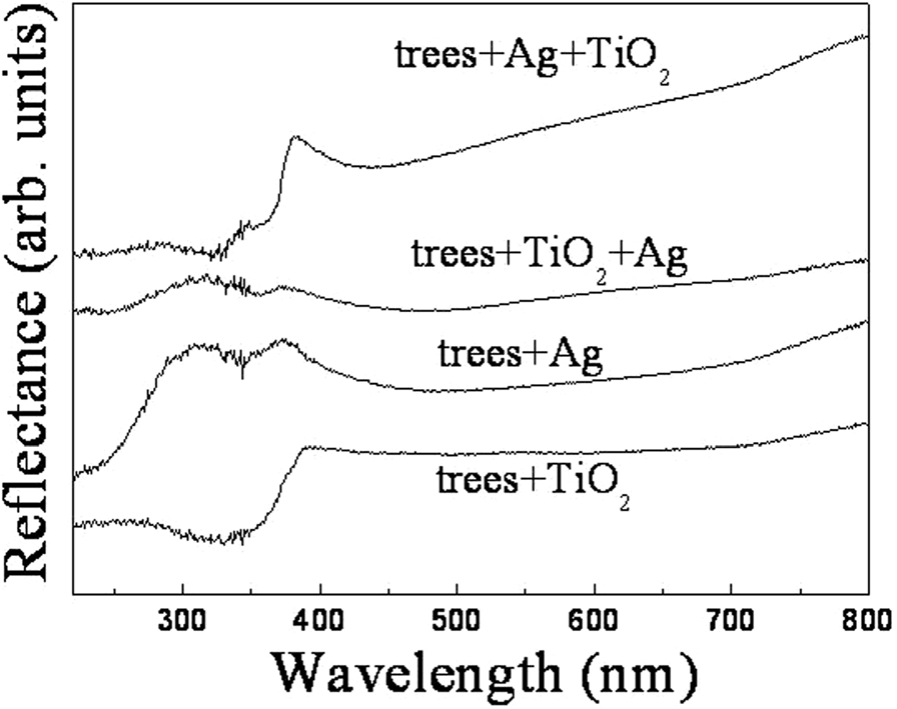
Diffuse reflection spectra of sample ALD with the coating layer. The spectra of trees in other coating layers were shifted upward in contrast to the spectrum of trees coated by TiO_2_ for comparison

To solve this dissolution problem and improve the photocatalytic efficiency, sample ALD was coated by a layer of Ag nanoparticles by solution photoreduction and a TiO_2_ film by ALD. The Ag nonoparticles on the surface of the nanowire arrays induce a Schottky junction between Ag and ZnO, which creates an internal electric field close to the interface and results in a decrease of the recombination rate of the photogenerated electrons and holes [28]. Moreover, the Ag nanoparticles exhibit localized surface plasmon resonance absorption of light which can significantly enhance semiconductor photocatalysis. On the other hand, TiO_2_ has been widely used as a protective layer for different semiconductor photoelectrodes due to its high resistance to corrosion and decomposition [2]. Figure 11 shows the SEM images of the hybrid nanowires coated by TiO_2_, Ag, Ag/TiO_2_, and TiO_2_/Ag, respectively. For comparison, the image of the pristine trees is also supplied in it. The reduced Ag particles exhibit a ball-like structure with the diameter in a range of 100–360 nm, as estimated from the amplified image of trees coated by Ag in Fig. 11f. The morphology of the samples in TiO_2_, Ag, and TiO_2_/Ag coating layers seems to stay the same as that of the pristine trees, suggesting the conformal ability of the coating methods. However, for the sample with Ag/TiO_2_ coating layer in Fig. 11d, the branches seem a bit denser than the others, which may be due to the observation direction with a smaller tilting angle. The coating layer of the sample is bigger than the others. They can be obviously seen with different structures. This is because the TiO_2_ layer is also deposited on the surface of the Ag particles. In this case, it provides a protective layer for the pristine trees as well as for the Ag particles. Figure 12 presents the reflection spectra of sample ALD with the coating layer. When the trees are coated by TiO_2_, the reflectance peak originating from the bandgap absorption gets a red shift. This is because the bandgap of TiO_2_ is smaller than that of ZnO. After the trees are coated by Ag particles, a broad band at ~315 nm arises, and the intensity of the characteristic peak of ZnO is reduced. The phenomenon can be ascribed to the presence of the resonance absorption of the Ag particles [28]. When the trees are coated by both Ag and TiO_2_, the band and the peak can be simultaneously observed, but their relative intensity is modulated by the relative position of the Ag and TiO_2_.

**Fig. 13:**
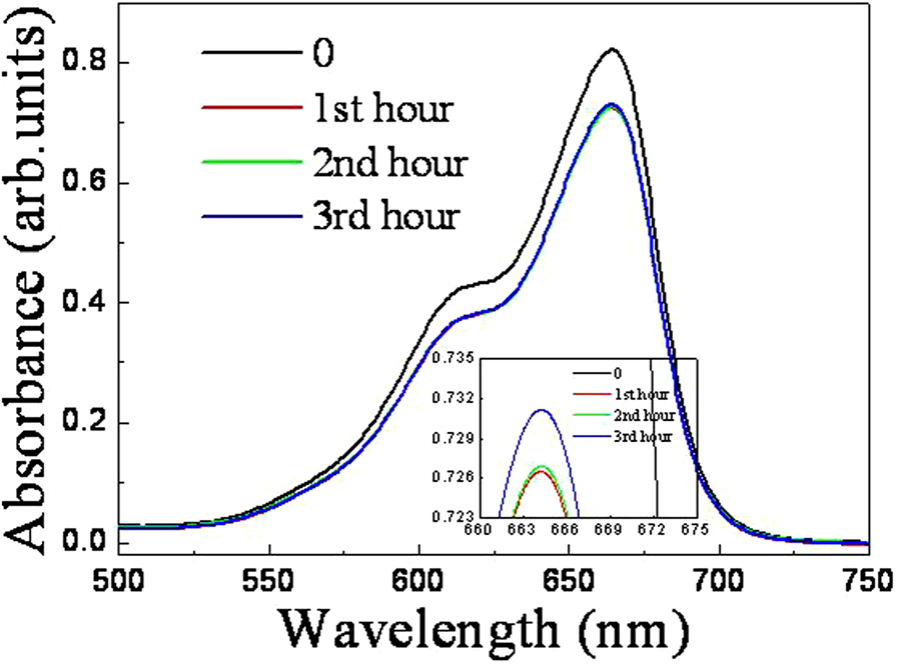
Absorption spectra of degraded MB aqueous solutions as catalyzed by sample ALD in Ag/TiO_2_ coating layer under irradiation of UV light, in which 0, 1st, 2nd, and 3rd hours represent spectrum of MB solution before photodegradation, after photodegradation in the 1st, 2nd, and 3rd hours, respectively. The *inset* shows the enlarged image of the absorption peak

We had examined photocatalytic activity of the hybrid nanowires in these coating layers and found that only the sample coated with Ag/TiO_2_ exhibited a stable photocatalytic performance. Figure 13 shows the absorption spectra of degraded MB solutions as catalyzed by sample ALD in the coating layer. The specimen presents a good recycling ability. The intensity of the absorption peak also increases for the latter degraded MB solution, but the change becomes very small. The acquired photocatalytic efficiency, as shown in Table 1, only decreases 0.56% in the 3 cycles. Nevertheless, in contrast to the efficiency of sample ALD without coating layer in the first hour, the efficiency of the specimen decreases about 4%, which may be assigned to the low conductivity and kinetic limitation of TiO_2_, as well as the shielding effect of the covered layer. The efficiency can be further improved by adjusting the diameter and length of both Si backbones and ZnO branches, the thickness of Ag layer and TiO_2_ layer, or selecting other noble metal and protective layer.

## Conclusions

ZnO/Si nanowire arrays with hierarchical structure were prepared by wet chemical etching and hydrothermal growth. The morphology, crystal structure, optical properties, and photocatalysis efficiency of the system were significantly influenced by the ZnO seed layer. In contrast to the seed layer deposited by MS, the more uniform seed layer deposited by ALD resulted in ZnO branches with better crystallinity grown on the whole sidewall of the Si backbones, in addition to the stronger light absorption and higher photodegradation efficiency of MB for its larger surface area and higher surface curvature effect. The photocatalytic efficiency of the system was declined under prolonging catalytic period, which might suffer from photodissolution and memory effect. A possible mechanism for the charge separation and organic dye pollutant degradation was proposed, and the stability and recycling ability of the system were improved by coating a layer of noble metal and metal oxide. All these suggested that the hybrid nanowires could find potential applications in photocatalysis and other fields, such as photovoltaics and sensors.

## Additional file

Additional file 1: Figure S1. High resolution XPS spectra of ZnO/Si nanowire arrays before and after photocatalysis. (a1–a3) Deconvolution of C (1s), O (1s), and Zn (2p) core levels in sample ALD before photocatalysis. (b1–b3) Deconvolution of C (1s), O (1s), and Zn (2p) core levels in sample ALD after photocatalysis. (c1–c3) Deconvolution of C (1s), O (1s), and Zn (2p) core levels in sample MS before photocatalysis. (d1–d3) Deconvolution of C (1s), O (1s), and Zn (2p) core levels in sample MS after photocatalysis. **Figure S2.** Spectral intensity of different bonds after photocatalysis (I) in contrast to that of before photocatalysis (I_0_) for sample ALD and sample MS as calculated from the deconvoluted spectra in **Figure S1.** (i) C-C bond, (ii) C-O-Zn bond, (iii) O-Zn bond, (iv) O-H bond or oxygen vacancies, (v) Zn 2p_3/2_, and (vi) Zn 2p_1/2_. (DOC 307 kb)
