# Supplementary material for: Synthesis of ZnO/Si Hierarchical Nanowire Arrays for Photocatalyst Application
Source: Nanoscale Res Lett. 2017 Jan 5;12:10. doi: 10.1186/s11671-016-1803-0 (PMC5216019; doi:10.1186/s11671-016-1803-0)
Supplement: Additional file 1: Figure S1. — High resolution XPS spectra of ZnO/Si nanowire arrays before and after photocatalysis. (a1–a3) Deconvolution of C (1s), O (1s), and Zn (2p) core levels in sample ALD before photocatalysis. (b1–b3) Deconvolution of C (1s), O (1s), and Zn (2p) core levels in sample ALD after photocatalysis. (c1–c3) Deconvolution of C (1s), O (1s), and Zn (2p) core levels in sample MS before photocatalysis. (d1–d3) Deconvolution of C (1s), O (1s), and Zn (2p) core levels in sample MS after photocatalysis. Figure S2. Spectral intensity of different bonds after photocatalysis (I) in contrast to that of before photocatalysis (I0) for sample ALD and sample MS as calculated from the deconvoluted spectra in Figure S1. (i) C-C bond, (ii) C-O-Zn bond, (iii) O-Zn bond, (iv) O-H bond or oxygen vacancies, (v) Zn 2p3/2, and (vi) Zn 2p1/2. (DOC 307 kb) [file 11671_2016_1803_MOESM1_ESM.doc]

**Synthesis of ZnO/Si Hierarchical Nanowire Arrays for Photocatalyst Application**

Dingguo Li^1,*^, Xiaolan Yan^1,*^, Chunhua Lin^2^, Shengli Huang1,2,3,4,[[1]](#footnote-2)a), Z. Ryan Tian^2^, Qianqian Yang^1^, Binbin Yu^1,3,4^, Xu He^1^, Jing Li^1^, Jiayuan Wang^1^, Huahan Zhan^1^, Shuping Li^1^, and Junyong Kang^1^

^1^ Fujian Provincial Key Laboratory of Semiconductors and Applications, Collaborative Innovation Center for Optoelectronic Semiconductors and Efficient Devices, Department of Physics, Xiamen University, Xiamen 361005, China

^2^ Department of Chemistry and Biochemistry, University of Arkansas, Fayetteville, AR 72701, USA

^3^ State Key Lab of Silicon Materials, Zhejiang University, Hangzhou 310027, China

^4^ Fujian Provincial Key Laboratory of Eco-Industrial Green Technology, Wuyi University, Fujian 354300, China

Figure S1 shows the deconvoluted XPS spectra of Sample ALD and Sample MS before and after photocatalytic performance. As the penetration depth of X-ray is not so far as that of electron in EDS, only 3 elemental species are detected on the surface of the samples, including C (1s), O (1s) and Zn (2p). Deconvolution of the C (1s) core level in Fig. S1 (a1) exhibits tow dominant peaks at 284.9 and 287.5 eV for Sample ALD before photocatalysis. According to the previous reseach [1, 2], the peak at 284.9 eV is ascribed to the presence of adventitious elemental carbon and C-C bond, and the peak at 287.5 eV is attributed to the C-O-Zn bond. The peak decomvolution of O (1s) exhibits two different peaks in Fig. S1 (a2). The first peak centered at 530.3 eV is assigned to the O-Zn bond in the ZnO wurtzite structure, the second peak at 531.3 eV is in accord with the O-H bond or oxygen vacancies on the surface of the sample. The XPS peaks in Fig. S1 (a3) at about 1022 and 1045 eV correspond to the Zn 2p_3/2_ and 2p_1/2_ states. Deconvolution of XPS spectra for other samples indicates identical elements with the bonds and states in the proximal binding energy.


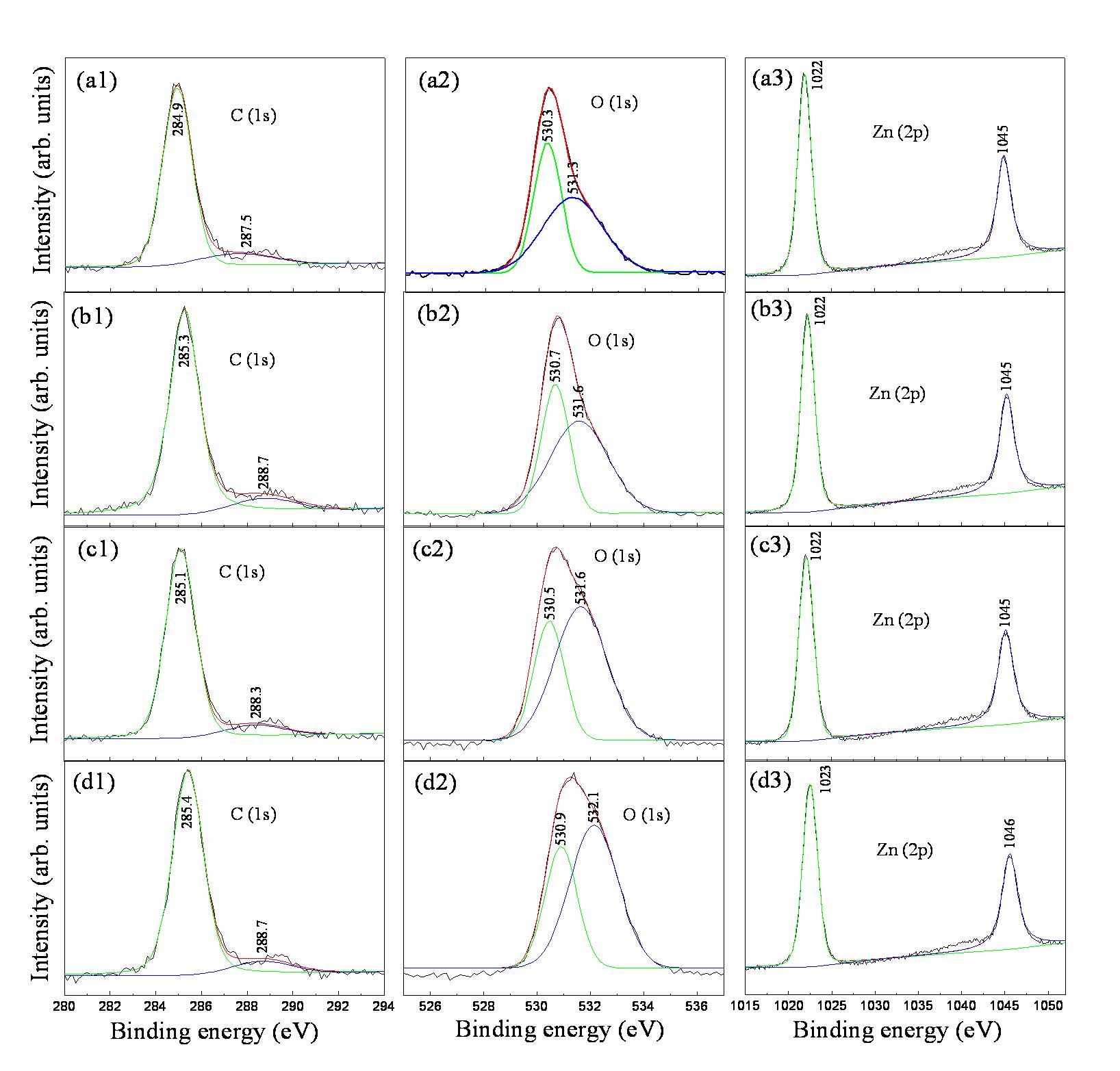


Figure S1. High resolution XPS spectra of ZnO/Si nanowire arrays before and after photocatalysis. (a1-a3): Deconvolution of C (1s), O (1s) and Zn (2p) core levels in Sample ALD before photocatalysis; (b1-b3): Deconvolution of C (1S), O (1S) and Zn (2P) core levels in Sample ALD after photocatalysis; (c1-c3): Deconvolution of C (1s), O (1s) and Zn (2p) core levels in Sample MS before photocatalysis; (d1-d3): Deconvolution of C (1s), O (1s) and Zn (2p) core levels in Sample MS after photocatalysis.

However, the intensity of the deconvoluted peaks, which indicates the amount of elemental species, changes a lot for different samples. Figure S2 shows the spectral intensity of different bonds after photocatalysis in contrast to that before photocatalysis for Sample ALD and Sample MS as caculated from the deconvoluted spectra in Figure S1. The peak intensity relating to Zn bonds of Sample ALD is reduced after the photocatalytic performance, while that of Sample MS becomes stronger. As the intensity responding to the amount of the binding bond, the decreasing intensity indicates the loss of ZnO in Sample ALD, whereas the enhancing intensity demonstrates the rise or stable state of ZnO in Sample MS.


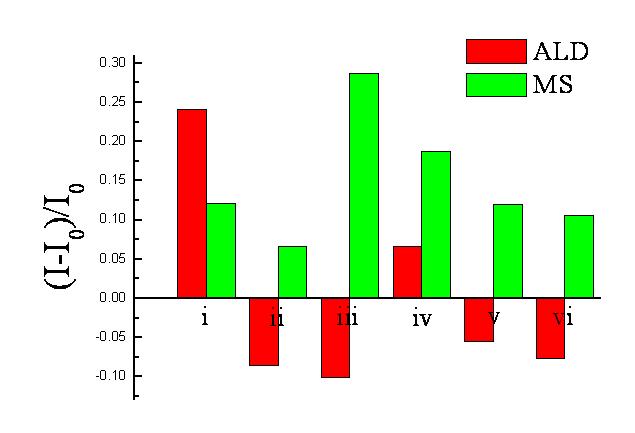


Figure S2. Spectral intensity of different bonds after photocatalysis (I) in contrast to that before photocatalysis (I_0_) for Sample ALD and Sample MS as caculated from the deconvoluted spectra in Figure S1: i) C-C bond, ii) C-O-Zn bond, iii) O-Zn bond, iv) O-H bond or oxygen vacancies, v) Zn 2p_3/2_, vi) Zn 2p_1/2_.

References

1. Samadi M, Shivaee H, Pourjavadi A, Moshfegh A (2013) [Synergism of Oxygen Vacancy and Carbonaceous Species on Enhanced Photocatalytic Activity of Electrospun ZnO-Carbon Nanofibers: Charge Carrier Scavengers Mechanism](http://www.sciencedirect.com/science/article/pii/S0926860X13003542). Appl Catal A-Gen 466:153– 160.

2. Sheng W, Sun B, Shi T, Tan X, Peng Z, Liao G (2014) Quantum Dot-Sensitized Hierarchical Micro/Nanowire Architecture for Photoelectrochemical Water Splitting. ACS Nano 8:7163–7169.

1. ^a^Corresponding author. E-mail: [huangsl@xmu.edu.cn](mailto:huangsl@xmu.edu.cn)

   * The authors contributed equally to this work. [↑](#footnote-ref-2)
